# Molecular and Phylogenetic Analyses of the Mediator Subunit Genes in *Solanum lycopersicum*

**DOI:** 10.3389/fgene.2019.01222

**Published:** 2019-11-27

**Authors:** Yunshu Wang, Honglian Liang, Guoping Chen, Changguang Liao, Yicong Wang, Zongli Hu, Qiaoli Xie

**Affiliations:** Laboratory of Molecular Biology of Tomato, Bioengineering College, Chongqing University, Chongqing, China

**Keywords:** Mediator complex subunits, *Solanum lycopersicum*, genomic characterization, expression analysis, plant stress response

## Abstract

The Mediator complex is a multi-subunit protein assembly that serves as a central scaffold to help regulate DNA-binding transcription factors (TFs) and RNA polymerase II (Pol II) activity controlled gene expression programmed in response to developmental or environmental factors. However, litter information about Mediator complex subunit (*MED*) genes in tomato is available, although it is an essential model plant. In this study, we retrieved 46 candidate *SlMED* genes from the genome of tomato, and a comprehensive analysis was conducted, including their phylogenetic relationship, chromosomal locations, gene structure, cis-regulatory elements prediction, as well as gene expression. The expression profiling of 46 *SlMED* genes was analyzed using publicly available RNA-seq data. Furthermore, we selected some *SlMED* genes to evaluate their expression patterns in various tissues and under different abiotic stress treatments by quantitative reverse transcription PCR experiments. This is the first detailed report to elucidate the molecular and phylogenetic features of the *MED* genes in tomato, and it provides valuable clues for further functional analysis in order to clarify the role of the *SlMED* genes in diverse plant growth, development and abiotic stress response.

## Introduction

In eukaryotes, transcription is primarily controlled by RNA polymerase II (Pol II) (Sikorski and Buratowski, 2009). Transcription by PolII is an intricate process that requires a large number of transcription factors (TFs), including DNA-binding TFs, general transcription factors (GTFs), as well as transcription coactivators (Davidson and Bolouri, 2002; Levine and Tjian, 2003). Mediator, a multi-subunit protein complex, is the central coactivator that acts as a bridge between DNA-binding TFs and the basal Pol II machinery assembled at the core promoter region (Kim et al., 1994; Kornberg, 2005; Borggrefe and Yue, 2011). The Mediator complex was originally identified and purified in *Saccharomyces cerevisiae* and was found to be required for the activator-dependent stimulation of transcriptional activation (Kelleher et al., 1990; Flanagan et al., 1991). Subsequent investigation showed that Mediator is evolutionarily conserved from yeast to higher organisms (Boube et al., 2002). The number of Mediator complex subunits can vary from 25 to 35 depending upon the species, and the yeast Mediator complex comprises 25 subunits. More than 30 different Mediator complex subunits have been described in different organisms, but only approximately 20 have been found in all eukaryotes (Conaway and Conaway, 2011b). Based on biochemical and structural studies, Mediator is organized into four separate domains (head, middle, tail, and a detachable kinase module). The primary function of the tail domain is its involvement in the interaction with the DNA-bound transcriptional regulators (TFs), and the head and the middle domains interact with the Pol II-TFIIF complex and C-terminal domain (CTD) of Pol II, respectively (Ansari and Morse, 2013). The evidence from genetic experiments suggests that the whole Mediator complex acts as a central component of the transcription machinery. Additionally, individual Mediator subunit also has significant gene-specific and even tissue-specific functions (Conaway and Conaway, 2011a).

In yeast and animals, Mediator subunits have been shown to have critical functions in cell and organismal viability (Tudor et al., 1999), embryonic viability (Ito et al., 2000; Gillmor et al., 2010; Risley et al., 2010), organ development (Rau et al., 2006), as well as human immunity and diseases (Spaeth et al., 2011). In plants, the research on the Mediator complex is relatively backward. In 2007, the Mediator complex of *Arabidopsis thaliana* has been successfully purified (Bäckström et al., 2007). Subsequently, some plant Mediator subunits (MED) have been functionally characterized and several plant *MED* genes have been demonstrated to have important regulatory roles in plant development, flowering, the regulation of hormone signaling pathways, and biotic and abiotic stress tolerance (Kidd et al., 2011). The Arabidopsis Mediator subunits, namely, AtMED12 and AtMED13, mediate the timing of embryo patterning (Gillmor et al., 2010); AtMED14 controls cell proliferation and shoot meristem development (Autran et al., 2002); AtMED25 regulates multiple pathways, such as hormone signaling pathways, flowering time, and abiotic and biotic stress responses (Yu and Lin, 2005; Kidd et al., 2009; Dorcafornell et al., 2011); AtMed17, AtMed18, and AtMed20 promote the transcription of miRNA and are responsible for the morphological development (Yun et al., 2011); AtMED8 functions in the production of root hairs (Sundaravelpandian et al., 2013); and AtMED16 affects iron homeostasis and is required for plant defence signaling crosstalk (Wathugala et al., 2012; Yan et al., 2014; Wang et al., 2015). Moreover, the functions of the Mediator subunits have also been established in other model plants. For instance, *OsMED4* has been proposed to be involved in regulating rice tiller growth (Li et al., 2008); *OsMED15* has been hypothesized to control seed development and seed size (Thakur et al., 2013); and *NtMed8* is a key regulator of tobacco vegetative development and floral organ size (Wang et al., 2011). Additionally, we found that *SlMED18* can regulate the development of leaves and stems in tomato (Wang et al., 2018).

Tomato (Solanum lycopersicum) is one of the most widely consumed vegetables and considered an essential model plant for studying plant development and fruit ripening (Consortium, 2012). Thus, a comprehensive analysis of all *SlMED* genes is really necessary. In this study, to advance our understanding of the evolution and functions of the *SlMED* genes, we investigated whole *SlMED* genes by using bioinformatic analyses and identified 46 *MED* genes in tomato. Further analysis of these genes’ structure, chromosome distribution, exon-intron organization, as well as the comprehensive protein sequence analysis and phylogenetic comparison were carried out. In order to reveal the expression pattern of *SlMED* genes in different organs and under various abiotic stress conditions, we obtained a pre-expression pattern of all of the *SlMED* genes and analyzed their regulatory promoter elements. Furthermore, the expression pattern of several *SlMED* genes was determined by quantitative real-time PCR analyses in different stages of plant development, especially in fruit ripening. We also characterized their expression under various abiotic stress conditions to confirm the reliability of our predicted results. These results provide details of the tomato Mediators, which will be useful for future cloning and the functional characterization of the *MED* genes.

## Methods

### Identification of SlMED Genes

In this study, a total of 46 MED proteins were identified by BLASTP search (e-value was set at 1e−5), and protein sequences of *Arabidopsis* and rice MED were used as queries to retrieve among the SGN (*Solanaceae* Genomics Network) (https://solgenomics.net/) and NCBI (National Center for Biotechnology Information) (http://www.ncbi.nlm.nih.gov/) database. The *A. thaliana* MED proteins were searched from the TAIR (The *Arabidopsis* Information Resource) database (http://www.arabidopsis.org/) and the protein information of rice (*Oryza sativa*) was downloaded from RGAP (Rice Genome Annotation Project) (http://rice.plantbiology.msu.edu/index.shtml), based on previous reports (Saloni et al., 2011; Dolan and Chapple, 2016). Putative MED proteins have been further identified by Hidden Markov Model (HMM) methods using each individual Med subunit domain. All candidate protein sequences were examined using PROSITE (http://expasy.org/tools/program.html) and SMART (http://smart.embl-heidelberg.de/) database for reliability. Full-length protein, DNA, and CDS (coding DNA sequence) sequence of *SlMED* were downloaded from the SGN. The molecular weight and isoelectric points of tomato MED proteins were detected by the ExPASy proteomics server (http://www.expasy.org/). 

### Protein Alignment and Phylogenetic Analysis

The full-length amino acid sequences of *Arabidopsis*, rice, and tomato were aligned using ClustalX 1.81 (http://www.clustal.org/) (Saitou, 1987). Unrooted phylogenetic trees were generated by MEGA5.02 program, in which the evolutionary history can be inferred by the neighbor-joining method. The best DNA/protein models we found was “JTT+G” and the Bootstrap analysis was performed using 1,000 replicates (Tamura et al., 2011; Bast, 2013).

### Gene Structure Analysis

The GSDS (Gene Structure Display Server program) software (http://gsds.cbi.pku.edu.cn) was used to reveal the exon/intron structures for individual tomato *MED* genes by comparison of CDS and corresponding genomic DNA sequences from SGN and NCBI (Hu et al., 2014).

### Chromosomal Localization

In order to determine the chromosomal locations of tomato *MED* genes, we obtained the starting and ending positions of all candidate genes on each tomato chromosome from the SGN and NCBI database. The physical map was drawn using the Tomato-EXPEN 2000 (https://solgenomics.net/cview/map.pl) and the resulting chromosome position of each candidate genes was indicated by gene name.

### Digital Gene Expression Analysis

For the digital expression profiles of *SlMED* genes, the standardized expression levels of candidate genes, based on the RNA-seq data, were downloaded from wild species LA1589 (*Solanum pimpinellifolium*) date in digital expression (RNA-seq) dataset of TFGD (the Tomato Functional Genomics) (http://ted.bti.cornell.edu/cgi-bin/TFGD/digital/home.cgi) database.

Moreover, the data of Tomato Lab (http://tomatolab.cshl.edu/∼lippmanlab2/allexp_query.html) and Tomato eFP Browser (http://bar.utoronto.ca/efp_tomato/cgi-bin/efpWeb.cgi) were used to further validate the expression pattern of *SlMED*. Gene expression levels in the different tissues were calculated according to RPKM (reads per kilobase million) values of RNA-seq data. The RPKM values were displayed in **Supplementary Table S2**. A heat map was generated using MEV 4.9.0 (multiple experiment viewer) (http://www.tm4.org/mev.html) to visualize the expression profiles of the *MED* genes in different tomato organs (Howe et al., 2010; Howe et al., 2011).

### Promoter cis-Acting Regulatory Element Analysis

The promoter sequences (3,000 bp upstream 5’UTR region) were searched from NCBI database using CDS of *SlMED* genes as the queries. Then the PlantCare database (http://bioinformatics.psb.ugent.be/webtools/plantcare/html/) was used to find the cis-acting regulatory elements of different hormones and stress-responsive in *SlMED* genes.

### Plant Material and Growth Conditions

In this experiment, AC^++^ (*S.* lycopersicum, “Ailsa Craig” AC^++^) (Peralta et al., 2005), near to isogenic lines containing *rin* (*ripening inhibitor*) (Knapp et al., 1989) and *Nr* (*Never-ripe*) (Thompson et al., 1999) were cultivated in a greenhouse with long day conditions (16-h light/25°C, 8-h dark/18°C). Tissues from roots, stems, leaves, sepals, flowers, and fruits of different stages were gathered from adult tomato plants (70-day-old tomato). The flowers were marked at anthesis and the ripening periods of fruits were recorded according to days post-anthesis (DPA). Tomato fruit ripening stages were divided from green fruits to ripe fruits, including IMG (immature green), MG (mature green), B (breaker), B+4 (4 days after breaker), and B+7 (7 days after breaker). After tissue collection, we frozen all plant samples in liquid nitrogen quickly and then stored at −80°C until RNA isolation.

### Plant Hormone and Stress Treatments

For the plant hormone and stress treatment, AC^++^ seeds were germinated with seedlings cultivated in the greenhouse (Peralta et al., 2005). Then, we selected the uniform potted 35-day-old tomato seedlings for diverse treatments. For the dehydration stress, the seedlings were carefully pulled out from the soil and cleaned cautiously using water until the soil was removed. Then they were put on dry filter paper at 25 ± 1°C and the leaves were harvested at different periods (Pan et al., 2012). For hormone treatment, the plant seedlings were sprayed, respectively, with H_2_O, 100 µM ABA, 50 µM MeJA, 100 µM ACC, and 50 µM GA3 solution (Zhu et al., 2014), enclosed in plastic instantly after spraying and treated after 0, 1, 2, 4, 8, 12, 24 h. Then the leaves of seedlings were gathered for study. All tissues were frozen in liquid nitrogen immediately, then stored at −80°C for the experiment.

### Extraction of Total Ribonucleic Acid and Analysis of Quantitative Real-Time Polymerase Chain Reaction

Total RNA was extracted from all mentioned plant tissues in this article using TRIzol reagent (Invitrogen, Shanghai, China). After DNase digestion (Promega), cDNA was synthesized with oligo(dT)20 as primer by RNA that reverse-transcribed using Moloney murine leukemia virus reverse transcriptase (Promega). The final tube of 10 µl quantitative reverse transcription PCR (qRT-PCR) reaction system included 1 µl cDNA as template, 0.25 µl 10 mM each primer, 5 µl 2× Go-Taq^®^ qPCR Master Mix (Promega, Beijing, China), and 3.5 µl distilled water. qRT-PCR was accomplished using the CFX96™ Real-Time System (Bio-Rad, USA) under the following conditions: 95°C 2 min, 40 cycles of 95°C 15 s, followed by 60°C 35 s and a melting curve was created and analyzed. The *SlCAC* gene was selected as an internal standard to quantitate the expression of *SlMED* genes. (Expósito-Rodríguez et al., 2008). All qRT-PCR reaction system also includes NTC (no template control) and NRT (no reverse transcription control). The analysis of the genes relative expression levels was directed using the 2^-∆∆C^ method (Livak and Schmittgen, 2001). All primers we used were designed by the Primer 5.0 software and showed in the **Supplementary Table S1**. Experiments were implemented independently with samples of biological triplicates. The data were analyzed by Origin 8.0 software.

### Statistical Analyses

The mean values of data were calculated from three replicates and presented as mean ± standard deviation. The t-test (**P < 0.01 and *P < 0.05) was used to analyze the significant differences. The Origin 8.0 software was used to perform the data analysis.

## Results

### Identification of the Mediator Complex Subunit Genes in Tomato

To identify the *MED* genes in tomato, keyword and BLAST searches were performed against the SGN and NCBI databases. A total of 46 MED proteins were identified. For convenience, the tomato *MED* genes were named based on the NCBI database (**Table 1**). Further analysis indicated that the coding region length was in the range of 359 (*SlMED11*) to 6,789 bp (*SlMED12*), which encoded polypeptides that varied from 90 aa with a molecular weight of 16.4 kDa to 2,262 aa with a molecular weight of 250.6 kDa. SlMED36 had the highest isoelectric point (10.12), while SlMED21 had the lowest isoelectric point (4.46). Detailed information for the *MED* genes in tomato, including their names, accession numbers, molecular details, their homologous protein in *Arabidopsis* as well as Mediator modules, are listed in **Table 1**.

**Table 1:** Overview of Mediator complex subunit genes identified in tomato. List of predicted genes and related information include sequenced ID, molecular details, their homologous protein in Arabidopsis thaliana, as well as Mediator modules.

Gene name	Accession number	Coding region length (bp)	Protein			Arabidopsis homologous	Accession number	E-value	Mediator Module
			Length (aa)	MW (Da)	pI				
*SlMED2/32*	Solyc08g078240.2	519	172	18,495.62	4.57	*AtMED2/32*	At1g11760	1e−64	Tail
*SlMED3/27a*	Solyc01g087230.2	1,236	411	45,129.97	7.25	*AtMED3/27*	At3g09180	2e−167	Tail
*SlMED3/27b*	Solyc05g047740.1	768	256	28,807.74	4.82	*–*	–	–	Tail
*SlMED4*	Solyc02g087180.2	1,275	425	46,404.32	5.06	*AtMED4*	At5g02850	3e−180	Middle
*SlMED5/33a*	Solyc01g080200.3	3,957	1,318	143,986.90	6.65	*AtMED5a/33a*	At3g23590	0.0	Tail
*SlMED5/33b*	Solyc06g008960.2	4,011	1,336	145,367.17	6.89	*AtMED5a/33a*	At3g23590	0.0	Tail
*SlMED5/33c*	Solyc09g064780.2	3,966	1,321	142,416.94	7.02	*AtMED5a/33a*	At3g23590	0.0	Tail
*SlMED6*	Solyc03g121210.2	699	232	26,249.21	7.64	*AtMED6*	At3g21350	3e−99	Head
*SlMED7*	Solyc04g081430.2	507	168	19,541.44	7.95	*AtMED7a*	At5g03220	1e−86	Middle
*SlMED8*	Solyc03g123960.2	1,593	530	58,172.59	9.30	*AtMED8*	At2g03070	e−151	Head
*SlMED9*	Solyc02g069520.2	618	205	24,419.80	6.14	*AtMED9*	At1g55080	2e−33	Middle
*SlMED10*	Solyc08g065160.2	606	201	21,542.21	5.00	*AtMED10b*	At1g26665	2e−71	Middle
*SlMED11*	Solyc04g014700.4	359	90	10,690.19	5.08	*AtMED11*	At3g01435	2e−40	Head
*SlMED12*	Solyc01g094620.2	6,789	2,262	250,670.46	8.91	*AtMED12*	At4G00450	0.0	Kinase
*SlMED13*	Solyc04g039950.2	5,802	1,933	209,178.61	5.52	*AtMED13*	At1G55325	0.0	Kinase
*SlMED14*	Solyc01g097320.2	5,376	1,792	194,300.79	7.23	*AtMED14*	At3g04740	0.0	Middle
*SlMED15*	Solyc04g009500.2	1,344	576	61,587.46	8.69	*AtMED15a*	At1g15780	2e−126	Tail
*SlMED16*	Solyc02g078520.2	3,741	1,246	134,511.81	5.92	*AtMED16*	At4g04920	0.0	Tail
*SlMED17*	Solyc12g006650.1	1,992	663	74,312.22	5.73	*AtMED17*	At5g20170	0.0	Head
*SlMED18*	Solyc06g008010.2	651	216	23,158.83	6.23	*AtMED18*	At2g22370	3e−127	Head
*SlMED19a*	Solyc09g020010.2	588	195	22,410.34	9.29	*AtMED19a*	At5g12230	4e−52	Head
*SlMED19b*	Solyc06g082900.3.	663	220	25,461.72	9.66	*AtMED19a*	At5g12230	6e−47	Head
*SlMED19c*	Solyc06g007660.2	621	205	23,981.20	9.67	*AtMED19a*	At5g12230	6e−50	Head
*SlMED20a*	Solyc12g009090.3	672	222	25,602.14	5.89	*AtMED20a*	At2g28230	2e−119	Head
*SlMED20b*	Solyc10g049550.1	423	140	16,166.65	7.85	*AtMED20a*	At2g28230	2e−47	Head
*SlMED20c*	Solyc10g055160.1	558	185	21,397.94	8.64	*AtMED20a*	At2g28230	1e−82	Head
*SlMED20d*	Solyc10g054950.1	648	215	24,537.34	8.60	*AtMED20a*	At2g28230	9e−95	Head
*SlMED20e*	Solyc05g041170.1	669	222	25,620.34	5.88	*AtMED20a*	At2g28230	7e−101	Head
*SlMED21*	Solyc02g080430.2	417	138	15,098.96	4.46	*AtMED21*	At4g04780	1e−64	Middle
*SlMED22*	Solyc03g083300.2	474	157	17,007.00	5.09	*AtMED22a*	At1g16430	2e−52	Head
*SlMED23*	Solyc05g016440.3	3,036	1,011	113,478.67	7.21	*AtMED23*	At1g23230	0.0	Tail
*SlMED25a*	Solyc05g009710.4	1,484	471	51,790.15	8.95	*AtMED25*	At1g25540	2e−49	Tail
*SlMED25b*	Solyc12g070100.1	2,418	805	86,438.37	9.01	*AtMED25*	At1g25540	0.0	Tail
*SlMED26b*	Solyc10g080930.1	1,368	455	51,189.54	7.59	*AtMED26b*	At5g05140	1e−106	Middle
*SlMED26c*	Solyc11g005450.2	1,050	349	39,939.18	5.69	*AtMED26c*	At5g09850	8e−84	Middle
*SlMED28*	Solyc08g074670.2	423	140	16,430.89	5.58	*AtMED28*	At3g52860	2e−30	Head
*SlMED30*	Solyc08g006670.2	597	198	20,626.82	5.05	*AtMED30*	At5g63480	5e−30	Head
*SlMED31*	Solyc03g094030.2	588	196	22,457.61	9.39	*AtMED31*	At5g19910	5e−78	Middle
*SlMED34*	Solyc08g074600.3	2,130	708	52,459.91	8.72	*AtMED34*	At1g31360	0.0	Unknown
*SlMED35a*	Solyc11g044340.1	3,033	1,010	115,128.21	8.85	*AtMED35a*	At1g44910	0.0	Unknown
*SlMED35b*	Solyc07g022760.2	3,276	1,023	121,646.93	6.16	*AtMED35a*	At1g44910	0.0	Unknown
*SlMED35c*	Solyc04g008700.2	3,123	1,040	113,055.33	8.63	*AtMED35c*	At3g19840	0.0	Unknown
*SlMED36*	Solyc03g025270.2	945	314	32,980.61	10.12	*AtMED36*	At4g25630	8e−167	Unknown
*SlMED37*	Solyc01g099660.2	2,010	669	74,641.87	5.37	*AtMED37e*	At5g42020	0.0	Unknown
*SlCDK8*	Solyc12g027870.1	1,011	336	38,393.40	8.84	*AtCdk8*	At5g63610	0.0	Kinase
*SlCycC*	Solyc06g083260.3	753	250	29,256.93	6.30	*AtCycC*	At5g48630	2e−136	Kinase

### Phylogenetic Relationship of Mediator Complex Subunit Genes

**Figure 1:**
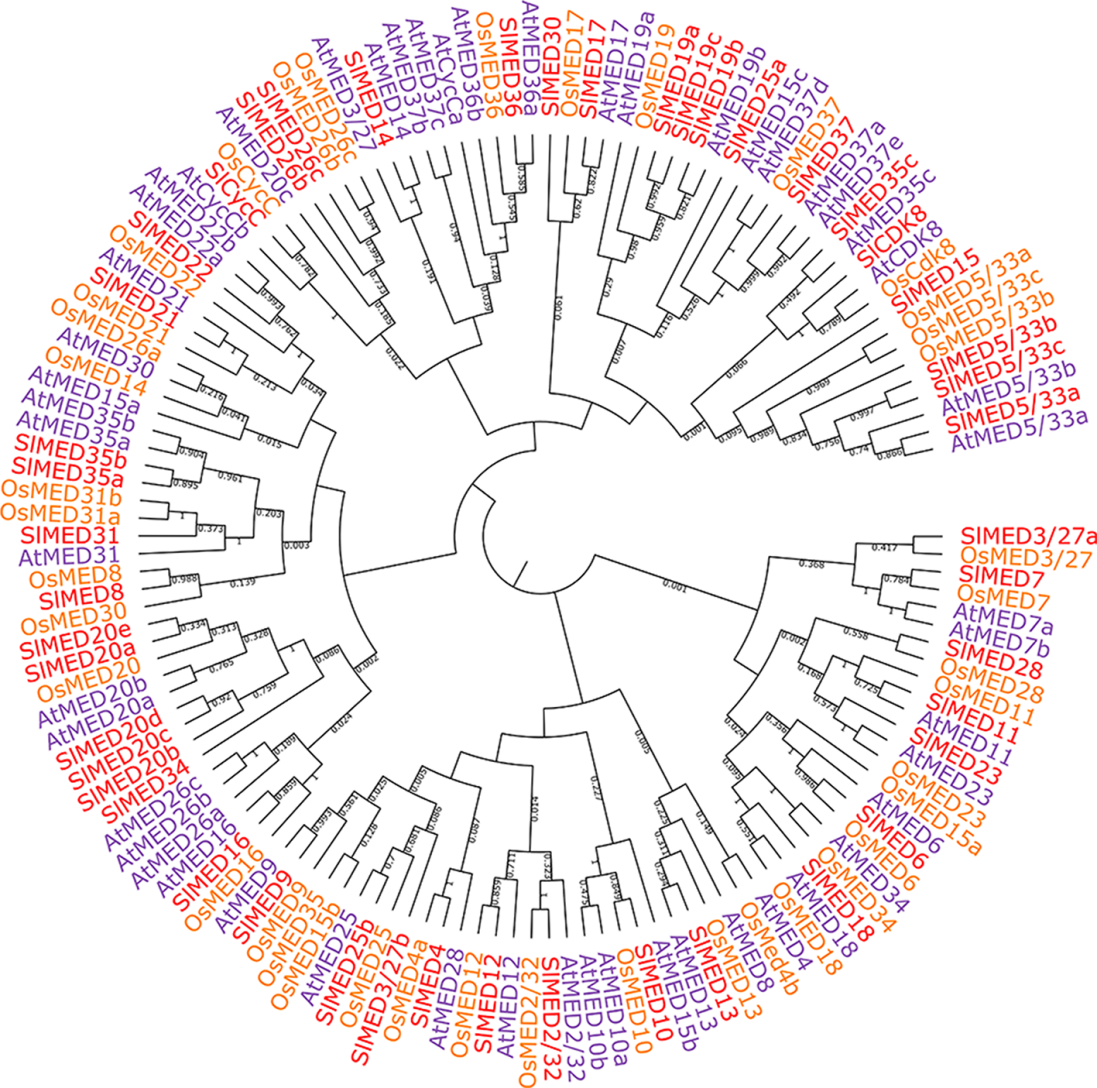
Phylogenetic relationship of Mediator subunits proteins between tomato, *Arabidopsis*, and rice. The phylogenetic tree was generated using MEGA 5.02 software and the neighbor-joining method with the following parameters: bootstrap analysis of 1,000 replicates, Poisson model, and pairwise deletion.

To analyze the phylogenetic relationships between tomato, Arabidopsis and rice MED proteins, multiple sequence alignments among 46 predicted tomato MED proteins, 49 *Arabidopsis* MED proteins, and 40 rice MED proteins were performed, and an N-J phylogenetic tree was constructed (**Figure 1**). The results illustrated that most of the MED proteins from tomato, *Arabidopsis*, and rice clustered with comparably high linkage on the phylogenetic tree such as SlMED6/AtMED6/OsMED6, SlMED17/AtMED17/SlMED17, SlMED18/AtMED18/OsMED18, and SlMED36/AtMED36/SlMED36. While the MED proteins among tomato clustered with little bootstrap support, and the MED proteins were not conserved across the same Mediator module. These results suggested close homology relationship and evolutionary conservation among MED proteins in plants.

### *Mediator Complex Subunit* Genes Structure and Chromosomal Location

**Figure 2:**
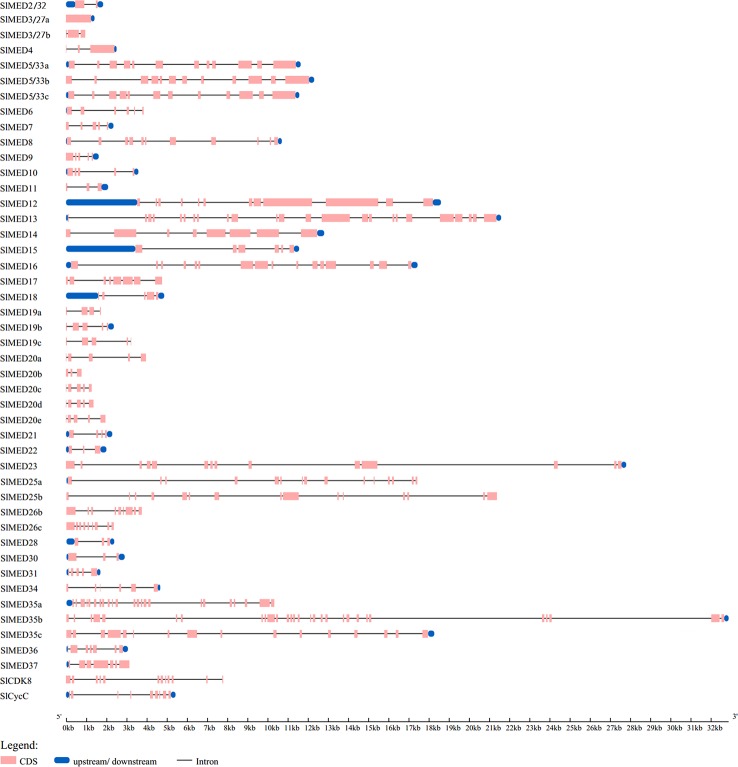
Gene structure analysis of Mediator subunits genes in tomato. The Gene Structure Display Server database was used to perform the exon-intron structure analyses. Lengths of exons and introns of each *SlMED* gene were displayed proportionally. The blue boxes represent upstream/downstream means untranslated region (UTR) including 5’-untranslated region (5’-UTR) and 3’-untranslated region (3’-UTR), the yellow boxes represent exons, and the black lines represent introns.

The exon-intron structure of all 46 *SlMED* genes was analyzed according to their genome sequences and corresponding coding sequences using the online tool GSDS (**Figure 2**). The result showed that the structure of these genes varied among members. The number of introns varied from 0 to 28. *SlMED3/27a* do not contain an intron in their genomic sequences, whereas *SlMED35b* has 28 introns. Consequently, the number of exons ranged from 1 to 29. Seven members have a simple structure with three exons. While most *SlMED* genes possess more than three exons.

**Figure 3:**
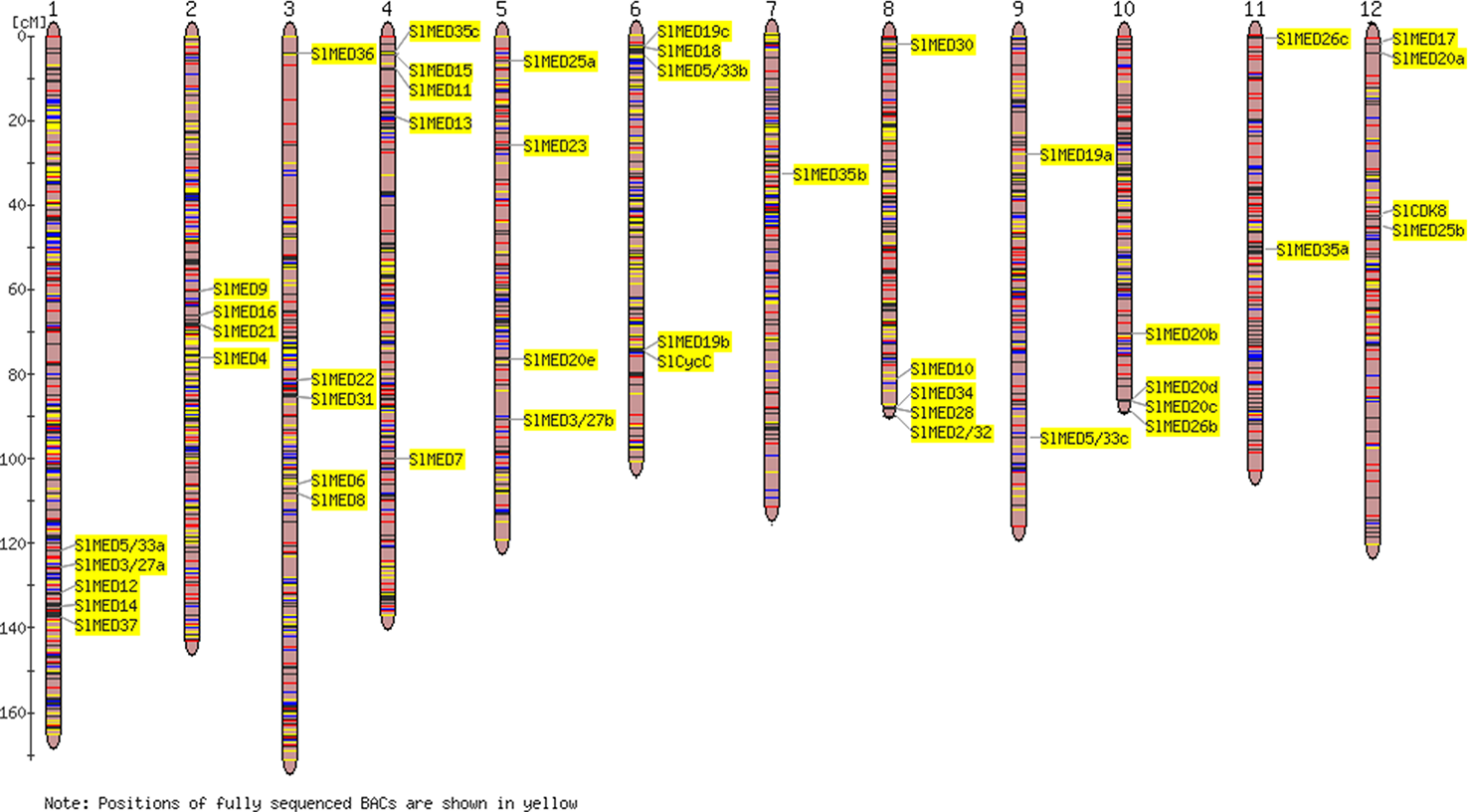
Chromosomal distributions of Mediator subunits (MED) genes in the tomato genome. Respective chromosome roman numbers are written at the top. The position of *SlMED* genes on the chromosome was based on *Solanaceae* Genomics Network and National Center for Biotechnology Information database and the Tomato-EXPEN 2000 tool was used to draw the physical map of the tomato *MED* genes.

To characterize the chromosomal distribution of the SlMED genes, the physical locations of 46 related genes on the tomato chromosomes were analyzed according to the genome sequencing information from the SGN database. As illustrated in **Figure 3**, *SlMED* genes were represented on almost all chromosomes. Furthermore, there were five *SlMED* genes distributed on chromosomes 1, 3 4, 6, and 8; four *SlMED* genes located on chromosomes 2, 5, 10, and 12; two *SlMED* genes mapped to chromosomes 9 and 11; and only one *SlMED* gene assigned to chromosome 7 (**Figure 3**).

### Ribonucleic Acid Sequencing Expression Analyses of *Mediator Complex Subunit* Genes in Tomato

**Figure 4:**
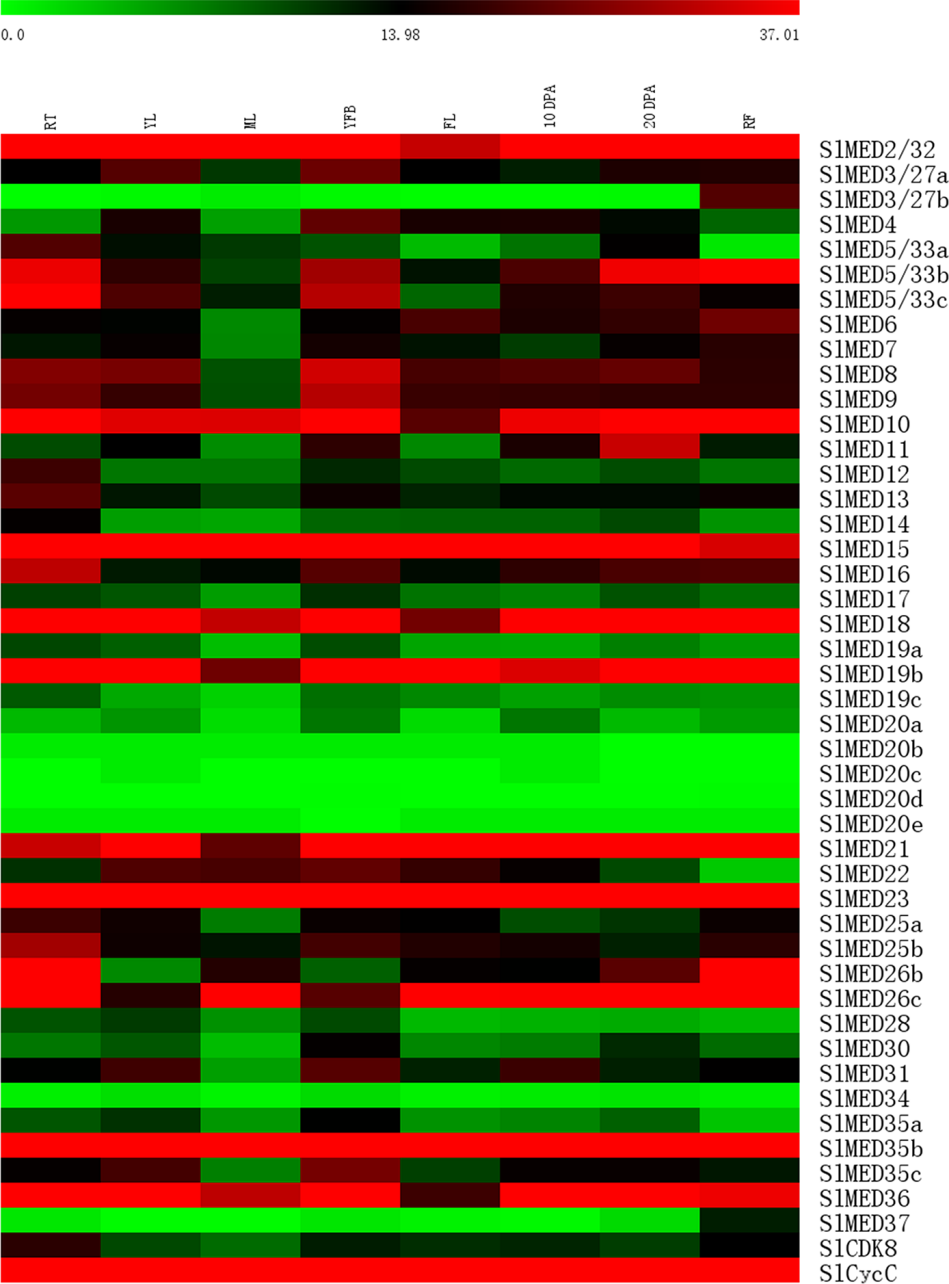
Heat map representation of tomato Mediator complex subunit genes in various tissues. The tissues included roots (RT), young leaves (YL), mature leaves (ML), young flower buds (YFB), fully open flowers (FL), 10 days post-anthesis fruits (10 DPA), 20 days post-anthesis fruits (20 DPA), and mature fruits (MF). The expression data was gained from Tomato Functional Genomics database and shown used MEV 4.9.0. The bar at the top of the heat map represents relative gene expression values.

Since high-throughput sequencing and gene transcription analyses have been conducted on various tomato tissues at different developmental stages, publicly available RNA-seq data are considered to be a useful resource for analyses gene expression profiles. Tomato transcript expression (RNA-seq) data in eight different tomato tissues, including roots (RT), young leaves (YL), mature leaves (ML), young flower buds (YFB), fully open flowers (FL), 10 days post-anthesis fruits (10 DPA), 20 days post-anthesis fruits (20 DPA), and mature fruits (MF), were used to investigate the expression profiles of *SlMED* genes. A hierarchical clustering heat map was constructed that displayed the expression patterns of 46 *SlMED* genes (**Figure 4**). Most of the *SlMED* genes exhibited distinct expression profiles across the eight tissues examined, indicating that different *SlMED* genes might function in diverse ways to regulate tomato growth and development. It is interesting to note that the expression levels of *SlMED2/32*, *SlMED15*, *SlMED23*, *SlMED35b*, and *SlCycC* were relatively higher in all eight tissues. However, three genes, *SlMED20b*, *SlMED20c*, *SlMED20d*, *SlMED20e*, and *SlMED34*, showed low levels of transcription in every organ tested. Specific *MED* genes, *SlMED3/27b* and SlMED37, were abundantly expressed in MF but had extremely lower transcript levels in other tissues. This finding suggested that these two genes might function in regulating tomato fruit growth and development.

### Promoter Structure Analysis of Tomato Mediator Complex Subunit Genes

**Figure 5:**
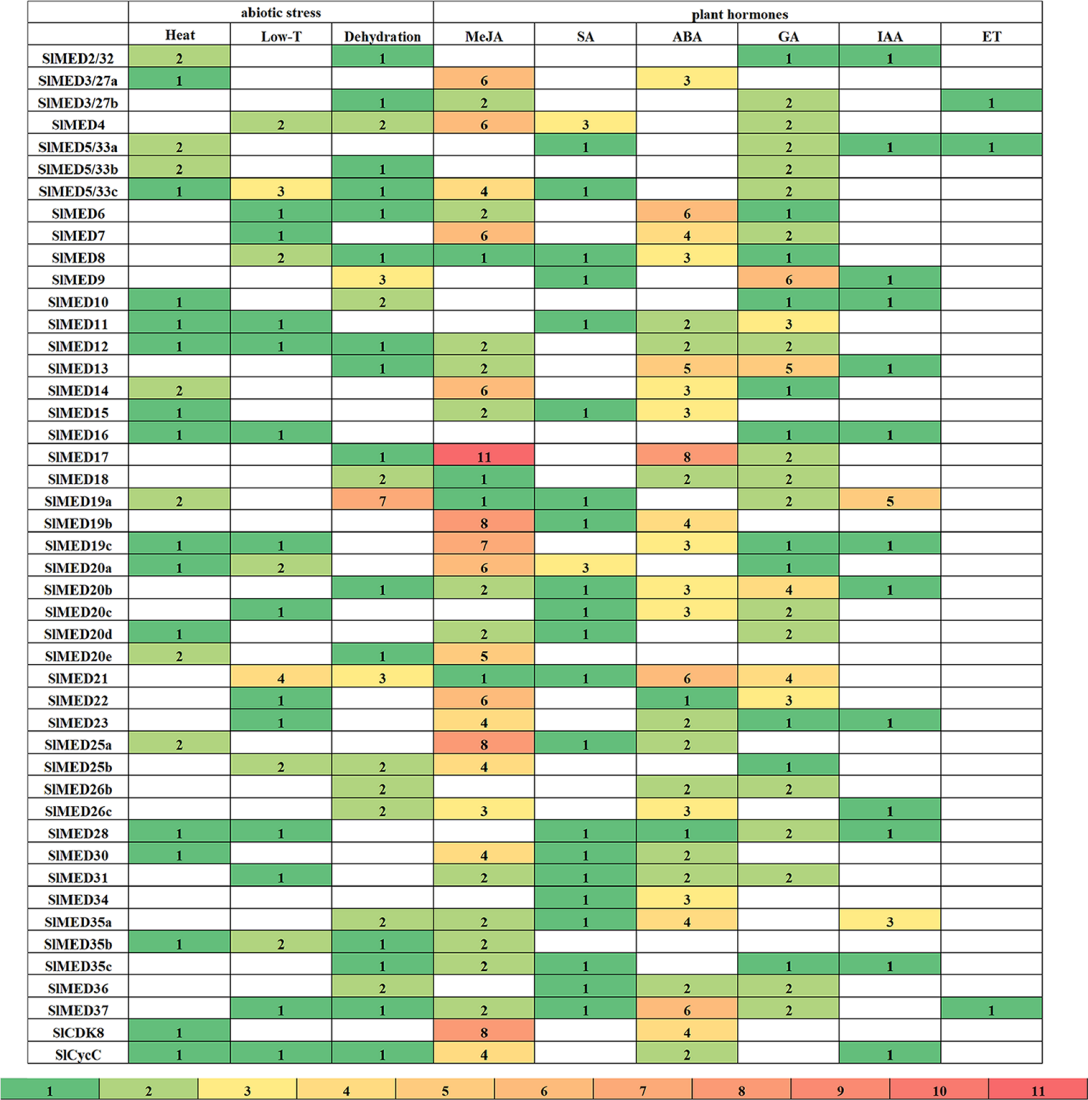
Numbers of stress related, hormone related regulatory elements in the upstream of *SlMED* genes. The cis-acting elements identified 3 kb upstream regions of the *SlMED* candidate genes. Different numbers are represented by diverse colors. MeJA, methyl jasmonate; SA, salicylic acid; ABA, abscisic acid; GA, gibberellin; IAA, auxin; ET ethylene.

The analysis of promoter structure is thought to be one of the most important ways to predict the promoter regions and expression profiles of a gene, as well as to reveal hidden transcriptional networks. In this study, we identified 3 kb promoter sequences of putative *SlMED* genes. Bioinformatic analyses of these sequences with the PlantCare database illuminated typical regulatory elements of plant gene promoters, containing elements that respond to phytohormone and abiotic stresses. The number of elements specifically responsive to plant hormones and abiotic stresses was identified in *SlMED* gene promoter regions as shown in **Figure 5**. Moreover, the names, position and their functional descriptions of each regulatory elements were listed in **Supplementary Table S3**. This analysis indicated that the promoter of each *SlMED* gene contained more than two important putative regulatory elements, implying that the expression of *SlMED* genes might be regulated by different phytohormones and abiotic stresses. For abiotic stresses, the regulatory elements for heat and dehydration stress were abundantly found in *SlMED* genes, while fewer elements for low temperature were identified. Additionally, several cis-acting motifs responsible for six kinds of phytohormones MeJA (methyl jasmonate), SA (salicylic acid), ABA (abscisic acid), GA (gibberellin), IAA (auxin), ET (ethylene), were also enriched. Most low-temperature responsive elements were discovered in the promoters of *SlMED21*. Seven regulatory elements required for dehydration were identified in the promoters of *SlMED19a*. Three were discovered in *SlMED9* and *SlMED21*. The promoters of *SlMED17*, *SlMED22*, and *SlMED23* contained a large number of regulatory elements necessary for MeJA, indicated they may play a role in MeJA stress response. *SlMED17* and *SlMED37* were observed to have more ABA-responsive elements in their promoters than the other *SlMED* genes. Moreover, six GA response elements were found in the promoter region of *SlMED9*, four were discovered in *SlMED20b*, three were found in *SlMED21*.

### Expression Profiles of Selected Tomato Mediator Complex Subunit Genes in Different Tissues by Quantitative Reverse Transcription Polymerase Chain Reaction

**Figure 6:**
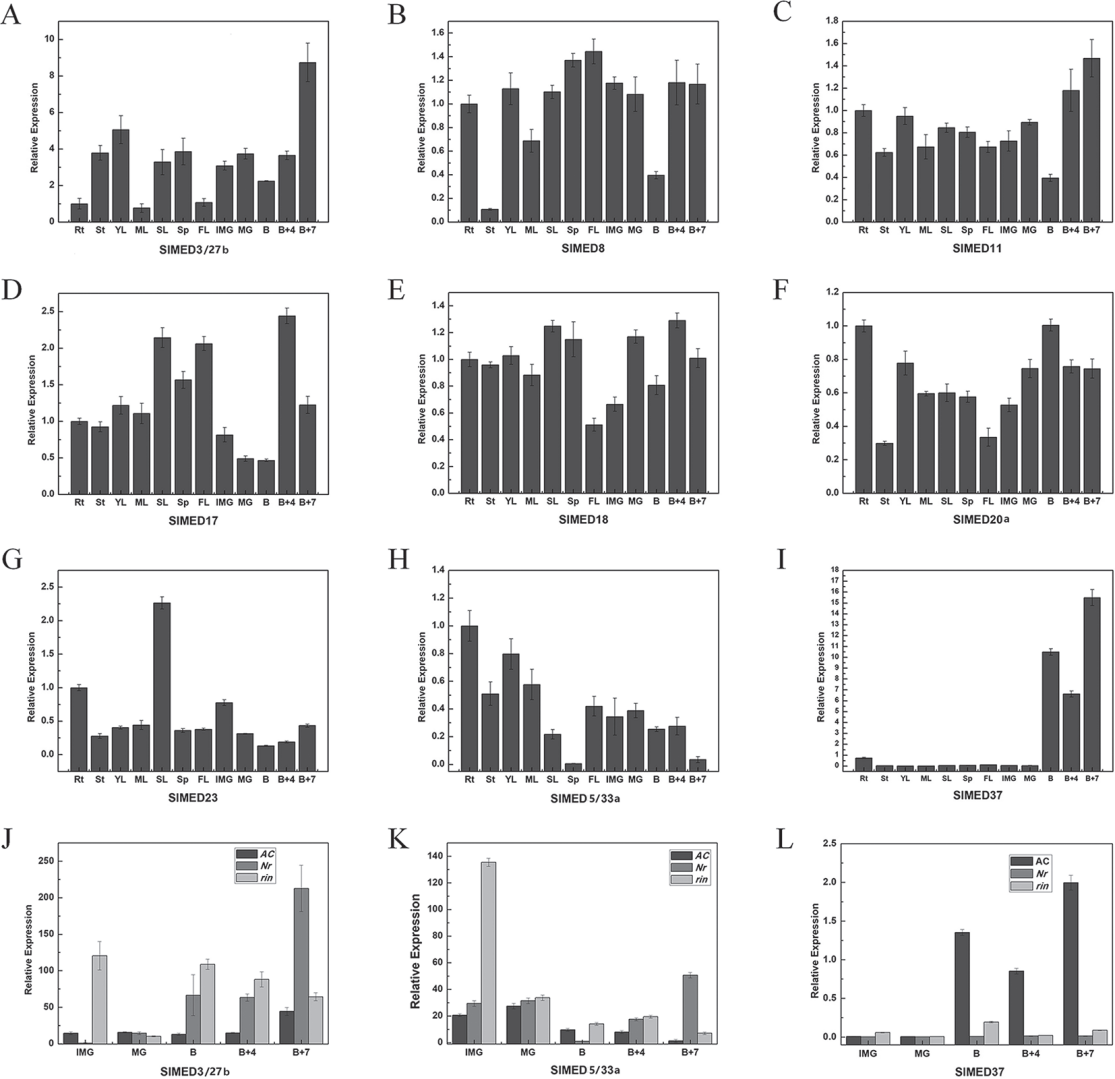
Quantitative PCR (qPCR) analysis of selected *SlMED* genes in different tissues. **(A**–**I)** Relative expression of selected *SlMED* genes in different developmental stages and tissues by qPCR analysis. The results were expressed using the root (Rt) as a reference for each gene (relative messenger RNA level 1). **(J**-**L)** Expression patterns of selected *SlMED* genes in wild type and ripening mutant fruits. The tissues included roots (RT), young leaves (YL), mature leaves (ML), senescent leaves (SL), sepal of flower in anthesis (SE), flowers (FL), immature green fruits (IMG), mature green fruits (MG), breaker fruits (B), 4 days after breaker fruits (B+4), and 7 days after breaker fruits (B+7). Each value represents the mean ± SD of three replicates.

According to the results of **Figure 4**, most tomato MED genes have abundant expression in diverse organs, except *SlMED3/27b*, *SlMED5/33a*, and *SlMED37* showed special expression trend during fruit development. To validate the gene expression profiles in diverse organs with the RNA-seq database, nine *SlMED* genes, including *SlMED3/27b*, *SlMED5/33a*, and *SlMED37*, were selected to confirm their expression in roots (RT), YL, ML, senescent leaves (SL), sepal of flower in anthesis (SE), flowers (FL), immature green fruits (IMG), mature green fruits (MG), breaker fruits (B), 4 days after breaker fruits (B+4), and 7 days after breaker fruits (B+7), using quantitative RT-PCR (**Figures 6A–I**). As was expected, most genes showed highly similar expression profiles between the RNA-seq date and qRT-PCR data. *SlMED11* was more highly expressed in 20 DPA according to the RNA-seq data, whereas the qRT-PCR data indicated that it was more highly expressed in B+7 (**Figure 6C**). Otherwise, the RNA-Seq expression patterns showed that SlMED17 expression level was higher in RT and YFB, which was inconsistent with the qRT-PCR data that displayed the highest expression level in B+7 (**Figure 6D**). These conflicting results between our RNA-seq data and qRT-PCR data might be due to the difference of plants growth conditions and experimental conditions. From these results, it can be speculated that some *SlMED* genes with different expression levels across multiple tomato tissues may have unique functions in plant specific development.

Notably, *SlMED3/27b* and *SlMED37* showed organ-preferential expression, which was expressed specifically and strongly in MF (**Figures 6A, I**). However, the expression of *SlMED5/33a* gradually decreased during the fruit growth and ripening stages (**Figure 6H**). Thus, further analysis of the transcriptional accumulation of *SlMED3/27b*, *SlMED5/33a*, and *SlMED37* from the IMG to B + 7 stages in normal *Nr* mutant and *rin* mutant fruits was conducted to investigate whether or not these three genes are related to the ripening-deficient mutants (**Figures 6J–L**). In wild type fruits and the *Nr* mutant, the expression of *SlMED3/27b* subsequently increased during fruit ripening and showed the highest level at the B+7 stage, while in the rin mutant the expression of *SlMED3/27b* indicated a decreased trend during fruit ripening (**Figure 6J**). A dissimilar expression trend was shown among the wild type, *Nr* and rin fruits of *SlMED5/33a*, indicating that *SlMED5/33a* expression may be impacted by *RIN* and *Nr* (**Figure 6K**). Additionally, *SlMED37* expression was found at a high level in B, B+4, and B+7 fruits in wild type, whereas its expression in *Nr* and *rin* was at nearly the same level during five stages of fruit development and ripening (**Figure 6L**). These data suggested that *SlMED37* may play a significant role in fruit ripening.

### Expression Pattern of Tomato Mediator Complex Subunit Genes Under Various Phytohormones and Abiotic Stresses

**Figure 7:**
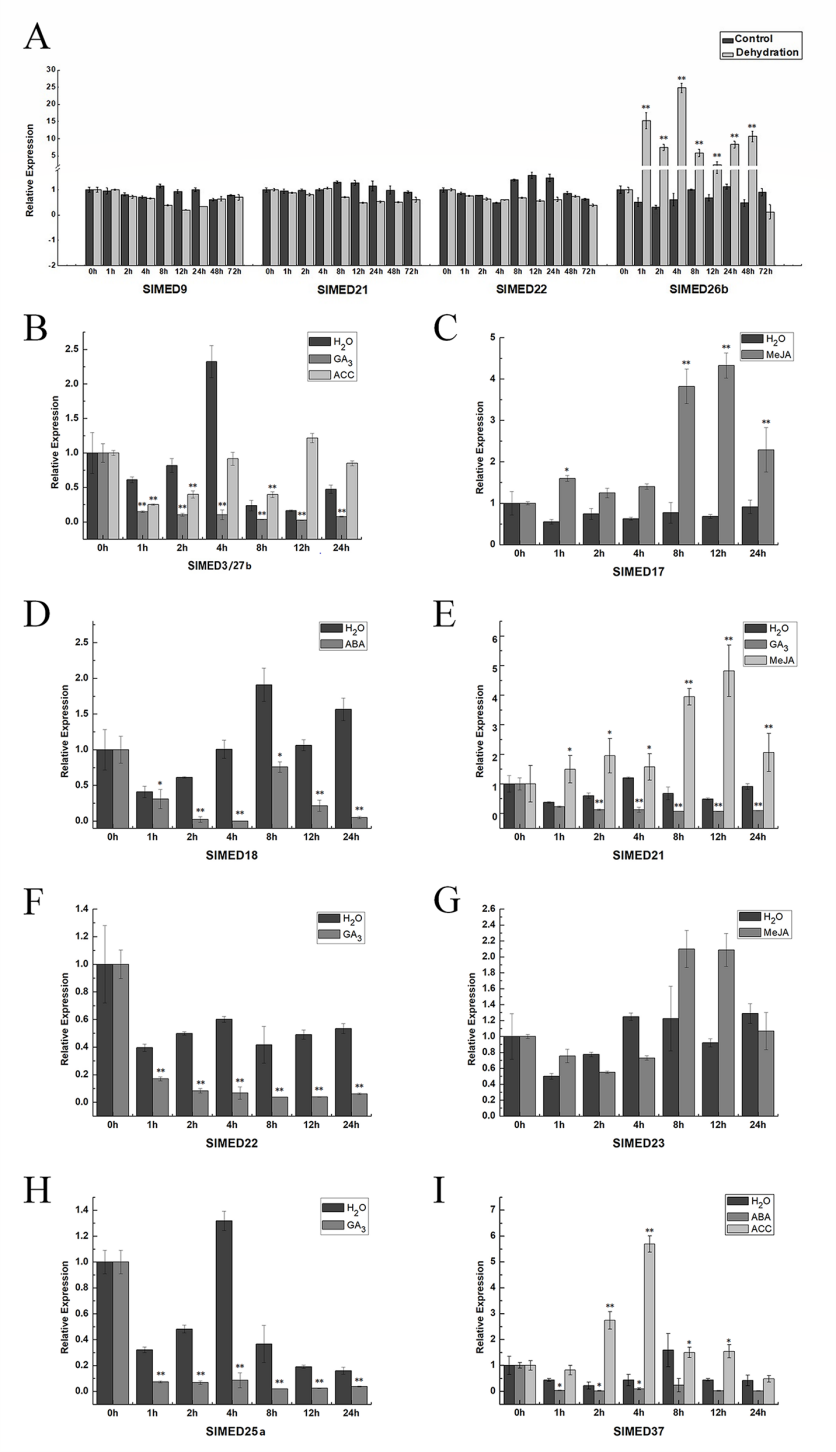
Expression patterns of the *SlMED* genes in response abiotic stress treatments. **(A)** Effect of dehydration on the expression of *SlMED9*, *SlMED21*, *SlMED22*, and *SlMED26b* genes in leaves by quantitative PCR analysis. **(B**–**I)** Expression profiles of the *SlMED* genes after various phytohormones treatment. Each value represent the experiment among three independent biological repetitions. Bars indicate the SEM of three experimental repetition.

From the promoter structure analysis of *SlMED* genes, we found these genes may have potential roles in response to various abiotic stresses. To gain more insight into the response and regulation of *SlMED* genes under abiotic stresses and hormone treatment, we selected some *SlMED* genes with a large number of regulatory elements in their promoter sequences and observed the expression profiles of these genes under dehydration, MeJA, ABA, GA, IAA, and ACC treatments using qRT-PCR (**Figure 7**). The expression levels of most of the genes examined changed greatly following exposure to these treatments. As shown in **Figure 7A**, *SlMED9*, *SlMED21*, and *SlMED22* had no significant changes in response to dehydration stress, while the expression level of *SlMED26b* was dramatically upregulated by dehydration stress (**Figure 7A**). Under the GA_3_ treatment, *SlED3/27b*, *SlMED21*, *SlMED22*, and *SlMED25a* were expressed at relatively low levels (**Figures 7B, E, F, H**). For ACC treatment, the level of *SlED3/27b* first declined, but after 4-h treatment, its expression returned to its original level (**Figure 7B**). In contrast, *SlMED37* was markedly upregulated at 2 and 4 h (**Figure 7I**). Interestingly, we found that *SlMED17*, *SlMED21*, and *SlMED23* were upregulated more than two-fold in response to MeJA after 8-h treatment (**Figures 7C, E, G**). The *SlMED18* gene expression decreased remarkably following ABA treatment (**Figure 7D**). However, there was no significant expression level change in *SlMED37* following ABA stress treatment (**Figure 7I**).

## Discussion

### Characterization of *Mediator Complex Subunit* Genes in Tomato

The Mediator complex serves an essential function in gene regulation, acting as a bridge between DNA-bound TFs and Pol II initiation machinery. Recently, reports revealed not only that the Mediator complex could regulate TFs expression but also that the individual Mediator subunit may have specific roles in plant development and abiotic stress responses (Hentges, 2011). In plants, molecular and phylogenetic analyses of *MED* genes have only been performed in *Arabidopsis* and rice (Bäckström et al., 2007; Mathur and Tyagi, 2011). Nevertheless, little is known about *MED* genes in tomato.

The comprehensive identification of the evolution, structure, and expression of *SlMED* genes provides new insight into their potential role. In this study, we first identified 46 *SlMED* genes through a genome-wide analysis, indicating that the numbers of tomato *MED* gene members were contracted compared to *Arabidopsis* (49) (**Table 1**). In tomato, the number of MED7, MED10, MED15, MED22, MED26, MED37, and CycC homolog proteins were less than *Arabidopsis*, whereas the tomato genome has more *MED19*, *MED20*, and *MED3/27* homolog genes. The loss of MED genes during evolution suggests that the function of some MED genes may be replaced by their homolog genes. And it is possible that more *MED19*, *MED20*, and *MED3/27* homolog genes are needed in tomato genome. In the phylogenetic tree, most tomato *MED* genes closely related to the *Arabidopsis* and rice homologues, indicating that the *MED* genes of tomato, *Arabidopsis*, and rice may have shared a close evolutionary relationship (**Figure 1**). This result was similar to previous reports that *MED* genes are conserved across the plant kingdom (Mathur and Tyagi, 2011). Some individual Mediator subunits have several homologue genes such as *SlMED19a*, *SlMED19b*, and *SlMED19c* sharing evolutionary origins which may play similar physiological functions. Additionally, the *SlMED* genes have various numbers of exons, implying diversity present in genes structure among *SlMED* genes. We found that several *SlMED* genes, such as *SlMED23* and *SlMED35b*, have long introns (**Figure 2**). These findings indicate structural differences and diversity in the *SlMED* genes.

In the plant kingdom, some reports have proven that *MED* genes function in multiple stages of plant development (Kidd et al., 2011; Lai et al., 2014a; Samanta and Thakur, 2015). In Mediator complex, the requirement for individual MED subunits varies. Some subunits are essential elements of Mediator architecture which are broadly required for Mediator function, while others only function in specific organs or pathways. Analysis of the tissue specific expression of *SlMED* genes is useful for evaluating their underlying biological functions in different organs. As a result, tomato transcript expression (RNA-seq) data were used to reveal the expression profiles of *SlMED* genes. We found that among the 46 predicted genes, most genes were highly expressed in all of the tissues tested, whereas *SlED3/27b* and *SlMED37* were specifically expressed in fruit ripening stages (**Figure 4**). In tomato, we found five MED20 paralogs (*SlMED20a*, *SlMED20b*, *SlMED20c*, *SlMED20d*, *SlMED20e*) which is more than in *Arabidopsis*. The transcript expression (RNA-seq) data showed that only *SlMED20a* had higher relative expression levels than other four paralogs. It is possible that *SlMED20a* plays a major role in tomato. The *SlMED20b*, *SlMED20c*, *SlMED20d*, and *SlMED20e* may have a function at some special growth stages or under particular environmental conditions. To further validate the expression profiles in different tissues gained from RNA-seq database, the expression of nine *SlMED* genes was detected by qRT-PCR in roots (RT), YL, ML, senescent leaves (SL), sepal of flower in anthesis (SE), flowers (FL), IMG, mature green fruits (MG), breaker fruits (B), 4 days after breaker fruits (B + 4), and 7 days after breaker fruits (B+7) (**Figure 6**). Furthermore, the statistical correlation (R value) between the relative expression values of the qRT-PCR results and the log2 RPKM values from the RNA-seq analysis were calculated and they were compared well, except for two genes (*SlMED11* and *SlMED17*) (**Supplementary Table S2**). The conflicting results between qRT-PCR date and RNA-seq data might be due to differences in plant materials, growth conditions, and experimental conditions. Both RNA-seq data and qRT-PCR result showed that *SlMED18* was abundantly expressed in all the tissues we examined. In our previous report, we found that the repression of *SlMED18* caused multiple plant developmental defects, and it was involved in the regulation of numerous plant growth and development processes in tomato, which was consistent with the expression profiles (Wang et al., 2018). These results may indicate the reliability of RNA-Seq expression patterns.

### Potential Functions of *SlMED* Genes During Fruit Development

Tomato ripening is a complex and highly coordinated developmental process associated with various physiological and biochemical modifications such as changes in colour, flavour, sugar, organic acids, as well as nutrient composition (Klee and Giovannoni, 2011). This process requires the activity of a series of TFs. The central coactivator complex, the Mediator complex, which acts as a bridge to transfer the message between TFs and the basal Pol II machinery assembled at the core promoter region, may perform essential roles in the regulation of fruit ripening. It was noteworthy that the expression levels of *SlED3/27b* and SlMED37 were very high in fruit after B stage, while *SlMED5/33a* exhibited gradually decreased expression during the fruit growth and ripening stages (**Figures 6A, H, I**). To date, a quantity of ripening-deficient mutants, such as the *never-ripe* (*Nr*) and ripening inhibitor (*rin*) mutants have been identified and studied extensively in tomato. The *Nr* mutant is insensitive to ET and shows an incomplete and delayed ripening phenotype (Rick, 1956; Lanahan et al., 1994; Hackett et al., 2000), and the *rin* mutant shows negative effects on all measured ripening phenomena, including carotenoid biosynthesis, increased respiration and ET production, flavor compound synthesis, and fruit softening (Tigchelaar et al., 1978; Kitagawa et al., 2005). To determine whether these three genes were related to tomato fruit development and ripening, we further analyzed the expression of *SlED3/27b*, *SlMED5/33a*, and *SlMED37* from the IMG to B + 7 stage in normal and ripening-deficient mutant (*Nr* and *rin*) fruits and discovered that the expression level of these three genes changed in different ripening-deficient mutants (**Figures 6J–L**). Thus, we speculate that *SlED3/27b*, *SlMED5/33a*, and *SlMED37* may be associated with the process of fruit development and ripening in tomato.

### Phytohormones and Abiotic Stress Responsive *Mediator Complex Subunit* Genes in Tomato

Plants perceive and integrate stress environmental signals, such as temperature, and dehydration, as well as various phytohormones, by different regulatory pathways. Several *Arabidopsis MED* genes have been shown to play key roles in the activation of stress signaling pathways. For example, *AtMED16*, *AtMED14*, and *AtMED2* were demonstrated to regulate COLD ON-REGULATED (COR) genes and they were insensitive to cold stress (Hemsley et al., 2014). *AtMED19a* was reported to be a key regulator in ABA-mediated transcriptional regulation (Li et al., 2018). In addition, *AtMED15* is known to act as a critical factor in the SA response (Canet et al., 2012).

The regulatory elements that are present in the promoter sequence, can bind a number of TFs and control gene regulation and expression. Observation of the promoter structure can support information on gene regulatory networks. According to promoter profiling analysis, we selected several *SlMED* genes with a large number of specific regulatory elements in the promoter sequences and investigated the expression profiles of these genes under various stress treatments using qRT-PCR. Dehydration is one of the most severe abiotic stress factors and is harmful to crop productivity. In this study, promoter profiling showed that two MBS (MYB binding site involved in drought-inducibility) elements, required for dehydration, were found in the promoters of *SlMED26b*. The MBS element is a general component in drought stress response genes (Abe et al., 1997). The response of plants to drought stress is likely to be dependent on the presence of such elements in specific gene promoters, so we speculated that *SlMED26b* might be a dehydration stress related gene in tomato. Additionally, qRT-PCR indicated that the expression level of *SlMED26b* was dramatically upregulated by dehydration stress (**Figure 7A**). This results also indicated that *SlMED26b* may be involved in the regulation of plant dehydration tolerance, potentially representing a new discovery of *MED* genes involved in dehydration stress. In addition, plant hormones are also well known to function in the regulation of plant growth and development. In view of the expression level of *SlED3/27b* after GA_3_ and ACC treatments together with the analyses of the regulatory elements, we propose that *SlED3/27b* may function as a regulator of GA_3_ and ACC signaling (**Figure 7B**). *AtMED17*, *AtMED21*, and *AtMED23* have been reported to play important roles in plant growth (Kidd et al., 2011). In tomato, these genes were upregulated in response to MeJA treatment, suggesting that they play potential roles in the resistance to MeJA stress (**Figures 7C, E, G**). Additionally, *SlMED21* and *SlMED22* showed reduced accumulation of mRNA under GA_3_ treatment, suggesting that negative control mechanisms might be present (**Figures 7E, F**). The expression level of *SlMED18* was decreased remarkably by ABA treatment, which is consistent with the report in which the Arabidopsis med18 mutant showed ABA insensitivity (Lai et al., 2014a). In *Arabidopsis*, it was found that *AtMED25* played a decisive role in JA signaling, whereas it had a negative effect on ABA signaling, which was also required for response to dehydration stress (Rong et al., 2012). Nevertheless, *SlMED25a* was induced by GA_3_ and contains four GA response elements in its promoter, implying that *SlMED25a* might have potential regulatory roles in GA_3_ stress responses (**Figure 7H**). *SlMED37*, a plant-specific Mediator subunit, was affected by ABA and ACC stresses in tomato (**Figure 7I**), suggesting their potential role under phytohormone stress. In particular, SlMED37 transcript level was remarkably increased by ACC and had the highest peak at 4 h. ACC is the immediate precursor of ET, and ET has been studied with respect to its critical roles in the ripening of fruit, the abscission of leaves, and abiotic stress adaptation. These results indicated that *SlMED37* might act as an essential factor during plant development and in response to abiotic stresses. In the future, we intend to focus on identifying the function of these cascade *SlMED* genes. For example, we will verify whether *SlMED26b* is related to the dehydration tolerance by constructing a SlMED26b overexpression vector and generating transgenic overexpression tomato plants to perform a dehydration stress treatment. In addition, performing *SlMED26b* gene mutagenesis with the CRISPR/Cas9 system transformation method may also prove to be a helpful strategy. Thus, our promoter structure analysis and expression profiles under various abiotic stressed provide a foundation to study the role to *SlMED* genes in resistance of abiotic stress.

## Conclusion

In conclusion, our study comprehensively performed a genome-wide analysis of tomato *MED* genes and provided systematic information about them. A total 46 of *SlMED* genes were identified, and their phylogenetic relationships with *Arabidopsis*, genomic organization, gene structure, cis-regulatory elements prediction, expression patterns among different tissues, and differential expression in response to phytohormones and abiotic stresses were characterized. In particular, *SlED3/27b*, *SlMED5/33a*, and *SlMED37* were found to might have a role in fruit development and ripening. Furthermore, we revealed the putative functions of *SlED3/27b*, *SlMED9*, *SlMED17*, *SlMED18*, *SlMED21*, *SlMED22*, *SlMED23*, *SlMED25a*, *SlMED26b*, and *SlMED37* in phytohormones and abiotic stress responses. These genes can be regarded as important candidates for further functional characterization. Taken together, our results will be helpful for obtaining a systematic understanding of *SlMED* genes and provide a useful reference for further functional studies of these genes.

## Data Availability Statement

The datasets generated for this study are available on request to the corresponding author.

## Author Contributions

GC, ZH and QX designed and managed the research work and improved the manuscript. YuW and YiW performed the bioinformatics analysis. YuW, HL and CL performed the experiments. YuW wrote the manuscript. All authors reviewed the manuscript.

## Funding

This work was supported by National Natural Science Foundation of China (nos. 30600044, 31572129) and the Training Program of Chongqing University Bioengineering College (0221001105301).

## Conflict of Interest

The authors declare that the research was conducted in the absence of any commercial or financial relationships that could be construed as a potential conflict of interest.

## References

[B1] Abe H. Yamaguchi-Shinozaki K. Urao T. Iwasaki T. Hosokawa D. Shinozaki K. (1997). Role of arabidopsis MYC and MYB homologs in drought- and abscisic acid-regulated gene expression. Plant Cell 9 (10), 1859–1868. 10.1105/tpc.9.10.1859 9368419 PMC157027

[B2] Ansari S. A. Morse R. H. (2013). Mechanisms of Mediator complex action in transcriptional activation. Cell. Mol. Life Sci. 70 (15), 2743–2756. 10.1007/s00018-013-1265-9 23361037 PMC11113466

[B3] Autran D. Jonak C. Belcram K. Beemster G. T. S. Kronenberger J. Grandjean O. (2002). Cell numbers and leaf development in Arabidopsis: a functional analysis of the STRUWWELPETER gene. EMBO J. 21 (22), 6036–6049. 10.1093/emboj/cdf614 12426376 PMC137206

[B4] Bäckström S. Elfving N. Nilsson R. Wingsle G. Björklund S. (2007). Purification of a plant mediator from Arabidopsis thaliana identifies PFT1 as the Med25 subunit. Mol. Cell 26 (5), 717. 10.1016/j.molcel.2007.05.007 17560376

[B5] Bast F. (2013). Sequence similarity search, multiple sequence alignment, model selection, distance matrix and phylogeny reconstruction. Nat. Protoc. 10.1038/protex.2013.065

[B6] Borggrefe T. Yue X. (2011). Interactions between subunits of the Mediator complex with gene-specific transcription factors. Semin. In Cell Dev. Biol. 22 (7), 759–768. 10.1016/j.semcdb.2011.07.022 21839847

[B7] Boube M. Joulia L. Cribbs D. L. Bourbon H. M. (2002). Evidence for a mediator of RNA polymerase II transcriptional regulation conserved from yeast to man. Cell 110 (2), 143–151. 10.1016/S0092-8674(02)00830-9 12150923

[B8] Canet J. V. Dobón A. Tornero P. (2012). Non-recognition-of-BTH4, an Arabidopsis mediator subunit homolog, is necessary for development and response to salicylic acid. Plant Cell 24 (10), 4220–4235. 10.1105/tpc.112.103028 23064321 PMC3517246

[B9] Conaway R. C. Conaway J. W. (2011a). Function and regulation of the Mediator complex. Curr. Opin. In Genet. Dev. 21 (2), 225–230. 10.1016/j.gde.2011.01.013 21330129 PMC3086004

[B10] Conaway R. C. Conaway J. W. (2011b). Origins and activity of the Mediator complex. Semin. In Cell Dev. Biol. 22 (7), 729–734. 10.1016/j.semcdb.2011.07.021 21821140 PMC3207015

[B11] Consortium T. T. G. (2012). ). The tomato genome sequence provides insights into fleshy fruit evolution. Nature 485 (7400), 635. 10.1038/nature11119 22660326 PMC3378239

[B12] Davidson E. H. Bolouri H. (2002). A genomic regulatory network for development. Science 295 (5560), 1669. 10.1126/science.1069883 11872831

[B13] Dolan W. L. Chapple C. (2016). Conservation and Divergence of Mediator Structure and Function: insights from Plants. Plant Cell Physiol. 58 (1), pcw176. 10.1093/pcp/pcw176 28173572

[B14] Dorcafornell C. Gregis V. Grandi V. Coupland G. Colombo L. Kater M. M. (2011). The Arabidopsis SOC1-like genes AGL42, AGL71 and AGL72 promote flowering in the shoot apical and axillary meristems. Plant J. 67 (6), 1006–1017. 10.1111/j.1365-313X.2011.04653.x 21609362

[B15] Expósito-Rodríguez M. Borges A. A. Borges-Pérez A. Pérez J. A. (2008). Selection of internal control genes for quantitative real-time RT-PCR studies during tomato development process. BMC Plant Biol. 8 (1), 131. 10.1186/1471-2229-8-131 19102748 PMC2629474

[B16] Flanagan P. M. Rd K. R. Sayre M. H. Tschochner H. Kornberg R. D. (1991). A mediator required for activation of RNA polymerase II transcription in vitro. Nature 350 (6317), 436–438. 10.1038/350436a0 2011193

[B17] Gillmor C. S. Park M. Y. Smith M. R. Pepitone R. Kerstetter R. A. Poethig R. S. (2010). The MED12-MED13 module of Mediator regulates the timing of embryo patterning in Arabidopsis. Development 137 (1), 113–122. 10.1242/dev.043174 20023166 PMC2796935

[B18] Hackett R. M. Chinwen H. Lin Z. F. Foote H. C. C. Fray R. G. Grierson D. (2000). Antisense inhibition of the Nr gene restores normal ripening to the tomato Never-ripe mutant, consistent with the ethylene receptor-inhibition model. Plant Physiol. 124 (3), 1079–1085. 10.1104/pp.124.3.1079 11080285 PMC59207

[B19] Hemsley P. A. Hurst C. H. Kaliyadasa E. Lamb R. Knight M. R. De Cothi E. A. (2014). The Arabidopsis mediator complex subunits MED16, MED14, and MED2 regulate mediator and RNA polymerase II recruitment to CBF-responsive cold-regulated genes. Plant Cell 26 (1), 465–484. 10.1105/tpc.113.117796 24415770 PMC3963590

[B20] Hentges K. E. (2011). Mediator complex proteins are required for diverse developmental processes. Semin. In Cell Dev. Biol. 22 (7), 769–775. 10.1016/j.semcdb.2011.07.025 21854862

[B21] Howe E. Holton K. Nair S. Schlauch D. Sinha R. Quackenbush J. (2010). Mev: MultiExperiment Viewer. Biomedical Informatics for Cancer Research Boston, MA: Springer 267–277. 10.1007/978-1-4419-5714-6_15

[B22] Howe E. A. Sinha R. Schlauch D. Quackenbush J. (2011). RNA-Seq analysis in MeV. Bioinformatics 27 (22), 3209. 10.1093/bioinformatics/btr490 21976420 PMC3208390

[B23] Hu B. Jin J. Guo A. Y. Zhang H. Luo J. Gao G. (2014). GSDS 2.0: an upgraded gene feature visualization server. Bioinformatics 31 (8), 1296. 10.1093/bioinformatics/btu817 25504850 PMC4393523

[B24] Ito M. Yuan C. X. Okano H. J. Darnell R. B. Roeder R. G. (2000). Involvement of the TRAP220 component of the TRAP/SMCC coactivator complex in embryonic development and thyroid hormone action. Mol. Cell 5 (4), 683. 10.1016/S1097-2765(00)80247-6 10882104

[B25] Kelleher R. J. Flanagan P. M. Kornberg R. D. (1990). A novel mediator between activator proteins and the RNA polymerase II transcription apparatus. Cell 61 (7), 1209–1215. 10.1016/0092-8674(90)90685-8 2163759

[B26] Kidd B. N. Edgar C. I. Kumar K. K. Aitken E. A. Schenk P. M. Manners J. M. (2009). The mediator complex subunit PFT1 is a key regulator of jasmonate-dependent defense in Arabidopsis. Plant Cell 21 (8), 2237–2252. 10.1105/tpc.109.066910 19671879 PMC2751954

[B27] Kidd B. N. Cahill D. M. Manners J. M. Schenk P. M. Kazan K. (2011). Diverse roles of the Mediator complex in plants. Semin. In Cell Dev. Biol. 22 (7), 741–748. 10.1016/j.semcdb.2011.07.012 21803167

[B28] Kim Y. J. Björklund S. Li Y. Sayre M. H. Kornberg R. D. (1994). A multiprotein mediator of transcriptional activation and its interaction with the C-terminal repeat domain of RNA polymerase II. Cell 77 (4), 599–608. 10.1016/0092-8674(94)90221-6 8187178

[B29] Kitagawa M. Ito H. Shiina T. Nakamura N. Inakuma T. Kasumi T. (2005). Characterization of tomato fruit ripening and analysis of gene expression in F1 hybrids of the ripening inhibitor (rin) mutant. Physiol. Plant. 123 (3), 331–338. 10.1111/j.1399-3054.2005.00460.x

[B30] Klee H. J. Giovannoni J. J. (2011). Genetics and control of tomato fruit ripening and quality attributes. Annu. Rev. Genet. 45 (1), 41. 10.1146/annurev-genet-110410-132507 22060040

[B31] Knapp J. Moureau P. Schuch W. Grierson D. (1989). Organization and expression of polygalacturonase and other ripening related genes in Ailsa Craig "Neverripe" and "Ripening inhibitor" tomato mutants. Plant Mol. Biol. 12 (1), 105–116. 10.1007/BF00017453 24272722

[B32] Kornberg R. D. (2005). Mediator and the mechanism of transcriptional activation. Trends In Biochem. Sci. 30 (5), 235–239. 10.1016/j.tibs.2005.03.011 15896740

[B33] Lai Z. Schluttenhofer C. M. Bhide K. Shreve J. Thimmapuram J. Lee S. Y. (2014a). MED18 interaction with distinct transcription factors regulates multiple plant functions. Nat. Commun. 5 (2), 3064. 10.1038/ncomms4064 24451981

[B34] Lanahan M. B. Yen H. C. Giovannoni J. J. Klee H. J. (1994). The never ripe mutation blocks ethylene perception in tomato. Plant Cell 6 (4), 521–530. 10.1105/tpc.6.4.521 8205003 PMC160455

[B35] Levine M. Tjian R. (2003). Transcription regulation and animal diversity. Nat. 424, 147–151. 10.1038/nature01763 12853946

[B36] Li Y. Zheng L. Corke F. Smith C. Bevan M. W. (2008). Control of final seed and organ size by the DA1 gene family in Arabidopsis thaliana. Genes Dev. 22 (10), 1331–1336. 10.1101/gad.463608 18483219 PMC2377187

[B37] Li X. Yang R. Gong Y. Chen H. (2018). The Arabidopsis Mediator Complex Subunit MED19a is Involved in ABI5-mediated ABA Responses. J. Plant Biol. 61 (2), 97–110. 10.1007/s12374-017-0277-7

[B38] Livak K. J. Schmittgen T. D. (2001). Analysis of relative gene expression data using real-time quantitative PCR and the 2 –ΔΔ C T method. Methods 25 (4), 402–408. 10.1006/meth.2001.1262 11846609

[B39] Mathur S. Tyagi A. K. (2011). The Mediator complex in plants: structure, phylogeny, and expression profiling of representative genes in a dicot (Arabidopsis) and a monocot (rice) during reproduction and abiotic stress. Plant Physiol. 157 (4), 1609–1627. 10.1104/pp.111.188300 22021418 PMC3327187

[B40] Pan Y. Seymour G. B. Lu C. Hu Z. Chen X. Chen G. (2012). An ethylene response factor (ERF5) promoting adaptation to drought and salt tolerance in tomato. Plant Cell Rep. 31 (2), 349–360. 10.1007/s00299-011-1170-3 22038370

[B41] Peralta I. E. Knapp S. Spooner D. M. (2005). New Species of Wild Tomatoes (Solanum Section Lycopersicon: Solanaceae) from Northern Peru. System. Bot. 30 (2), –. 10.1600/0363644054223657

[B42] Rau M. J. Fischer S. Neumann C. J. (2006). Zebrafish Trap230/Med12 is required as a coactivator for Sox9-dependent neural crest, cartilage and ear development. Dev. Biol. 296 (1), 83–93. 10.1016/j.ydbio.2006.04.437 16712834

[B43] Rick C. M. (1956). Phytogenetics of the tomato. Adv.Genet. 8, 267–382. 10.1016/S0065-2660(08)60504-0

[B44] Risley M. D. Clowes C. Yu M. Mitchell K. Hentges K. E. (2010). The Mediator complex protein Med31 is required for embryonic growth and cell proliferation during mammalian development. Dev. Biol. 342 (2), 146. 10.1016/j.ydbio.2010.03.019 20347762

[B45] Rong C. Hongling J. Lin L. Qingzhe Z. Linlin Q. Wenkun Z. (2012). The Arabidopsis mediator subunit MED25 differentially regulates jasmonate and abscisic acid signaling through interacting with the MYC2 and ABI5 transcription factors. Plant Cell 24 (7), 2898–2916. 10.1105/tpc.112.098277 22822206 PMC3426122

[B46] Saitou N. (1987). The neighbor-joining method : a new method for reconstructing phylogenetic tree. Mol. Biol. Evol. 4 (4), 406. 10.1093/oxfordjournals.molbev.a040454 3447015

[B47] Saloni M. Shailendra V. Sanjay K. Akhilesh Kumar T. (2011). The Mediator complex in plants: structure, phylogeny, and expression profiling of representative genes in a dicot (Arabidopsis) and a monocot (rice) during reproduction and abiotic stress. Plant Physiol. 157 (4), 1609–1627. 10.1104/pp.111.188300 22021418 PMC3327187

[B48] Samanta S. Thakur J. K. (2015). Importance of Mediator complex in the regulation and integration of diverse signaling pathways in plants. Front. In Plant Sci. 6 (7), 388–394. 10.3389/fpls.2015.00757 26442070 PMC4584954

[B49] Sikorski T. W. Buratowski S. (2009). The basal initiation machinery: beyond the general transcription factors. Curr. Opin. In Cell Biol. 21 (3), 344. 10.1016/j.ceb.2009.03.006 19411170 PMC2692371

[B50] Spaeth J. M. Kim N. H. Boyer T. G. (2011). Mediator and human disease. Semin. In Cell Dev. Biol. 22 (7), 776–787. 10.1016/j.semcdb.2011.07.024 21840410 PMC4100472

[B51] Sundaravelpandian K. Chandrika N. N. P. Schmidt W. (2013). PFT1, a transcriptional Mediator complex subunit, controls root hair differentiation through reactive oxygen species (ROS) distribution in Arabidopsis. New Phytol. 197 (1), 151–161. 10.1111/nph.12000 23106228

[B52] Tamura K. Peterson D. Peterson N. Stecher G. Nei M. Kumar S. (2011). MEGA5: molecular evolutionary genetics analysis using maximum likelihood, evolutionary distance, and maximum parsimony methods. Mol. Biol. Evol. 28 (10), 2731–2739. 10.1093/molbev/msr121 21546353 PMC3203626

[B53] Thakur J. K. Agarwal P. Parida S. Bajaj D. Pasrija R. (2013). Sequence and expression analyses of KIX domain proteins suggest their importance in seed development and determination of seed size in rice, and genome stability in Arabidopsis. Mol. Genet. Genomics Mgg 288 (7-8), 329–346. 10.1007/s00438-013-0753-9 23756993

[B54] Thompson A. J. Tor M. Barry C. S. Vrebalov J. Orfila C. Jarvis M. C. (1999). Molecular and genetic characterization of a novel pleiotropic tomato-ripening mutant. Plant Physiol. 120 (2), 383. 10.1104/pp.120.2.383 10364389 PMC59276

[B55] Tigchelaar E. C. Mcglasson W. B. Buescher R. W. (1978). Genetic regulation of tomato fruit ripening. Hortsci. A Publ. Am. Soc. Hortic. Sci. 13 (5), 508–513.

[B56] Tudor M. Murray P. J. Onufryk C. Jaenisch R. Young R. A. (1999). Ubiquitous expression and embryonic requirement for RNA polymerase II coactivator subunit Srb7 in mice. Genes Dev. 13 (18), 2365–2368. 10.1101/gad.13.18.2365 10500093 PMC317028

[B57] Wang F. Wei H. Tong Z. Zhang X. Yang Z. Lan T. (2011). Knockdown of NtMed8, a Med8-like gene, causes abnormal development of vegetative and floral organs in tobacco (Nicotiana tabacum L.). Plant Cell Rep. 30 (11), 2117–2129. 10.1007/s00299-011-1118-7 21744120

[B58] Wang C. Yao J. Du X. Zhang Y. Sun Y. Rollins J. A. (2015). The Arabidopsis Mediator Complex Subunit16 Is a Key Component of Basal Resistance against the Necrotrophic Fungal Pathogen Sclerotinia sclerotiorum. Plant Physiol. 169 (1), 856–872. 10.1104/pp.15.00351 26143252 PMC4577384

[B59] Wang Y. Hu Z. Zhang J. Yu X. Guo J. E. Liang H. (2018). SilencingSlMED18, tomato Mediator subunit 18 gene, restricts internode elongation and leaf expansion. Sci. Rep. 8 (1), 3285. 10.1038/s41598-018-21679-1 29459728 PMC5818486

[B60] Wathugala D. L. Hemsley P. A. Moffat C. S. Cremelie P. Knight M. R. Knight H. (2012). The Mediator subunit SFR6/MED16 controls defence gene expression mediated by salicylic acid and jasmonate responsive pathways. New Phytol. 195 (1), 217–230. 10.1111/j.1469-8137.2012.04138.x 22494141

[B61] Yan Y. Bin O. Jinzhe Z. Wen S. Hongya G. Genji Q. (2014). The Arabidopsis Mediator subunit MED16 regulates iron homeostasis by associating with EIN3/EIL1 through subunit MED25. Plant J. 77 (6), 838–851. 10.1111/tpj.12440 24456400

[B62] Yu X. Lin C. (2005). Light Regulation of Flowering Time in Arabidosis. Light Sensing in Plants. Tokyo: Springer Verlag 325–332. 10.1007/4-431-27092-2_38

[B63] Yun J. K. Zheng B. Yu Y. Won S. Y. Mo B. Chen X. (2011). The role of Mediator in small and long noncoding RNA production in Arabidopsis thaliana. EMBO J. 30 (5), 814–822. 10.1038/emboj.2011.3 21252857 PMC3049218

[B64] Zhu M. Hu Z. Zhou S. Wang L. Dong T. Pan Y. (2014). Molecular Characterization of Six Tissue-Specific or Stress-Inducible Genes of NAC Transcription Factor Family in Tomato ( Solanum lycopersicum). J. Plant Growth Regul. 33 (4), 730–744. 10.1007/s00344-014-9420-6

