# Supplementary material for: Molecular and Phylogenetic Analyses of the Mediator Subunit Genes in Solanum lycopersicum
Source: Front Genet. 2019 Nov 27;10:1222. doi: 10.3389/fgene.2019.01222 (PMC6892441; doi:10.3389/fgene.2019.01222)
Supplement: Supplementary file 1 [file Table_1.docx]

***Supplementary Material***

Molecular and Phylogenetic Analyses of the Mediator subunit genes in Solanum lycopersicum

Yunshu Wang^1^, Honglian Liang^1^, Guoping Chen^1^, Changguang Liao^1^, Yicong Wang^1^, Zongli Hu^1^* QiaoliXie^1^*

^1^ Laboratory of molecular biology of tomato, Bioengineering College, Chongqing University, Chongqing, People’s Republic of China

*** Correspondence:**

Zongli Hu

Bioengineering College, Chongqing University, Chongqing, People’s Republic of China

Phone: 00862365102507

Fax: 00862365102507

E-mail: [huzongli71@163.com](mailto:huzongli71@163.com)

Qiaoli Xie

Bioengineering College, Chongqing University, Chongqing, People’s Republic of China

Phone: 00862365102507

Fax: 00862365102507

E-mail, qiaolixie@cqu.edu.cn

**Co-authors:**

Yunshu Wang

E-mail: wangyunshu@cqu.edu.cn

Honglian Liang

E-mail: [20161902007t@cqu.edu.cn](mailto:20161902007t@cqu.edu.cn)

Guoping Chen

E-mail: [chenguoping@cqu.edu.cn](mailto:chenguoping@cqu.edu.cn)

Changguang Liao

liaochangguang@cqu.edu.cn

Yicong Wang

E-mail: luka-miku@outlook.com

**Supplementary Table S1.** Primers for PCR amplification and quantification. All the primers we used were designed by Primer premier 5.0 software.

| Primer names | Sequences (5' →3') |
| --- | --- |
| SLMED3-Q-F | ATGGTATGCGTGAATTTCCGA |
| SlMED3-Q-R  SlMED8-Q-F  SlMED8-Q-R | CTCATTTTGATAGTGACTGCCTGTT  CAACACCTATCGGTAGCATCCC  CCCCTGTCTGGTTCCAAAGTAG |
| SlMED9-Q-F | AAATGCTAAATCAGCGAAGGGA |
| SlMED9-Q-R  SlMED11-Q-F  SlMED11-Q-R | GACAGGTGATTCAACTCAACTTCTTT  TAATCAAGGACATCCAAGTCACACT  TAATCAAGGACATCCAAGTCACACT |
| SlMED15a-Q-F | GCCTTTGAGATTGCTCGTTG |
| SlMED15a-Q-R | AAGACTTCGCAGGGAGACACTA |
| SlMED17-Q-F | TTCTCAAACAAAGTCCTCGCAG |
| SlMED17-Q-R | ACATAGTCAGATGAGTGAGACAGGC |
| SlMED18-Q-F | ATATCTTGCACCGCTCCTTCA |
| SlMED18-Q-R | GGGAAGGAGAATGGCGAAATA |
| SlMED19a-Q-F | TGATGGAGAGGAGGAATTTAATGAT |
| SlMED19a-Q-R  SlMED21-Q-F  SlMED21-Q-R | GGGTGCTTGAGCAAAAAAGTTAG  GAGGGGAATAGTTATGGAGATGG  TGGTAATGATCGTTTTCCAAGAG |
| SlMED21-Q-F | TCCTTTATCGGATGGGAGTGAA |
| SlMED21-Q-R | CAGGCAGTTATCTGTTGCTTGGT |
| SlMED23-Q-F | CATTTCGCATTATGGGTCCC |
| SlMED23-Q-R  SlMED25-Q-F  SlMED25-Q-R | CCGTTGCTTCAATAGGTGCTG  GTATTTATTACCAGATTGGAGGGG  CGATGGGAGTTGTATTACCGC |
| SlMED26b-Q-F | ATGTTCGGCATCTATCTCGGAC |
| SlMED26b-Q-R | CTAAGTCATCTAAAGGCGGGG |
| SlMED33-Q-F | GCCATTTAATAGGCTACGCTCC |
| SlMED33-Q-R | GATATTGGGAGCACACGAACC |
| SlMED37-Q-F | AAGGTGAAAGGAGCTTAACGAA |
| SlMED37-Q-R  SlCAC-Q-F  SlCAC-Q-R | ATTTCTTCCTGGCTTAGACGC  CTCCGTTGTGATGTAACTGG  ATTGGTGGAAAGTAACATCATCG |

**Supplementary Table S2.**  The RPKM values of RNA- seq data for SlMED genes in the different tissues.

**Supplementary Table S3.** Kinds and numbers of stress‐related and hormone‐related, in the upstream of *SlMED* genes. The names, position and their functional descriptions of each regulatory elements were listed.
